# 4273 An innovative rib construct for treatment of pediatric spinal deformity

**DOI:** 10.1017/cts.2020.156

**Published:** 2020-07-29

**Authors:** Daniel Bonthius

**Affiliations:** 1 Medical University of South Carolina

## Abstract

OBJECTIVES/GOALS: The rib construct is a novel device for treating childhood hyperkyphosis and kyphoscoliosis. The purpose of this study was to investigate the biomechanics, mechanism, and clinical outcomes of this device. The overarching hypothesis was that the rib construct is safe and effective for correcting hyperkyphotic spinal deformity. METHODS/STUDY POPULATION: **Biomechanical evaluation:** An *ex vivo* porcine spine biomechanical study compared traditional pedicle screw proximal fixation to the rib construct in terms of proximal fixation strength and construct stiffness. **Porcine model hyperkyphosis correction with rib construct:** An *in vivo* hyperkyphotic porcine model was used to study the ability of the rib construct to correct hyperkyphosis in the developing porcine spine. **Human hyperkyphotic correction with rib construct:** A retrospective study was conducted to examine the radiographic outcomes, complication rates, procedure times, and blood losses experienced by human patients that received rib construct surgery. RESULTS/ANTICIPATED RESULTS: **Biomechanical evaluation:** The rib construct was significantly less prone to proximal fixation failure and less stiff compared to pedicle screws. **Porcine model hyperkyphosis correction with rib construct:** The average T6-T14 thoracic kyphosis was 35.8 ± 3.2° at the time of hyperkyphosis creation surgery. In response to corrective surgery with the rib-hook construct, T6-T14 thoracic hyperkyphosis decreased immediately post-op to 11.3 ± 7.8° and continued to decrease to 7.8 ± 7.6° until final follow-up 8 weeks post-op (n = 3). **Human hyperkyphosis correction with rib construct:** Pre-op sagittal Cobb angle was 81 ± 31° and fell to 43 ± 24° post-op and to 38 ± 24° at final follow-up; indicating ~100% correction (normal thoracic kyphosis is 40°). DISCUSSION/SIGNIFICANCE OF IMPACT: The results suggest that the rib construct is a highly effective technique and superior to existing methods.

